# Highly efficient synthesis of [60]fullerene oxides by plasma jet

**DOI:** 10.1098/rsos.170658

**Published:** 2017-09-06

**Authors:** Sheng-Peng Jiang, Shengxia Duan, Kai-Qing Liu, Xiao-Yu Yang, Cheng Cheng, Jiaxing Li, Guan-Wu Wang

**Affiliations:** 1 CAS Key Laboratory of Soft Matter Chemistry, Collaborative Innovation Center of Chemistry for Energy Materials, Hefei National Laboratory for Physical Sciences at Microscale and Department of Chemistry, University of Science and Technology of China, Hefei, Anhui 230026, People's Republic of China; 2 Institute of Plasma Physics, Chinese Academy of Sciences, PO Box 1126, Hefei 230031, People's Republic of China; 3 Collaborative Innovation Center of Radiation Medicine of Jiangsu Higher Education Institutions, Suzhou, Jiangsu, People's Republic of China

**Keywords:** plasma jet, C_60_O, C_60_O_2_, theoretical calculations

## Abstract

Atmospheric pressure nonequilibrium plasma jet has been applied to the synthesis of [60]fullerene oxides (C_60_O_n_) for the first time. C_60_O and C_60_O_2_ were produced and isolated in high yields up to 44% and 21%, respectively. The structural assignment of C_60_O was confirmed by comparison with the reported spectroscopic data. Theoretical calculations of ^13^C NMR chemical shifts for eight isomers of C_60_O_2_ were performed and compared with the experimental data to assign the most possible structure for the obtained C_60_O_2_ dominantly as an *e* isomer.

## 1. Introduction

Since the discovery of [60]fullerene (C_60_), various C_60_ derivatives have been synthesized due to their broad potential applications in materials science, biology and nanoscience [1–7]. Among these derivatives, the study of fullerene oxides (C_60_O_n_) has been a popular area of research for decades. Particularly, the monoxide C_60_O is the best characterized oxidation product of C_60_, and exhibits unique properties [8–11]. In 1992, Cox, Smith and co-workers reported the photochemical synthesis of C_60_O in 16% yield by UV irradiation of an oxygenated benzene solution of C_60_ in the presence of benzyl [12]. Nearly at the same time, Foote, Whetten and co-workers described the formation of C_60_O in 4% yield from the oxidation reaction of C_60_ with dimethyldioxirane [13]. In 1995, the Balch group disclosed that the oxidation reaction of C_60_ with *m*-chloroperoxybenzoic acid (*m*-CPBA) afforded C_60_O in 30% yield and C_60_O_2_ as the *cis*-1 isomer in 8% yield [14]. In addition, the formation of C_60_O and C_60_O_n_ (*n* ≥ 2) was identified by mass spectroscopy or high-performance liquid chromatography (HPLC), yet no isolated yields were reported in the oxidation of electrochemically generated anionic C_60_ [15] and ozonolysis of C_60_ [16–18]. Interestingly, C_60_O has been employed as the precursor for further functionalizations, leading to the formation of C_120_O [19], 1,3-dioxolane derivatives of C_60_ [20], indoline derivatives of C_60_ [21], and 1,2-perfluorophenylfullerenol [22]. Similarly, the *cis*-1 isomer of C_60_O_2_ has been used as the precursor for bis-1,3-dioxolane derivatives of C_60_ [20]. Despite these achievements, new approach to obtain C_60_O and C_60_O_2_ in higher yields is still highly demanding. Furthermore, the efficient synthesis and characterization of other isomeric C_60_O_2_ except *cis*-1 isomer are still unknown until now.

Recently, atmospheric pressure nonequilibrium plasma jet (APNPJ) has attracted extensive attention due to its wide range of applications in plasma biology, healthcare, medicine as well as surface and materials processing [23–28]. Because APNPJ generates plasma in open space rather than in confined gaps, there are no restrictions on the size of the objects to be treated, and short lifetime active species and even charged particles can easily reach the employed objects [29–33]. To the best of our knowledge, the application of APNPJ to fullerene chemistry has not been reported until now. Herein, we report the synthesis of C_60_O and C_60_O_2_ by APNPJ for the first time. This novel method features simple operation and high efficiency.

## 2. Experimental

**Figure 1.**
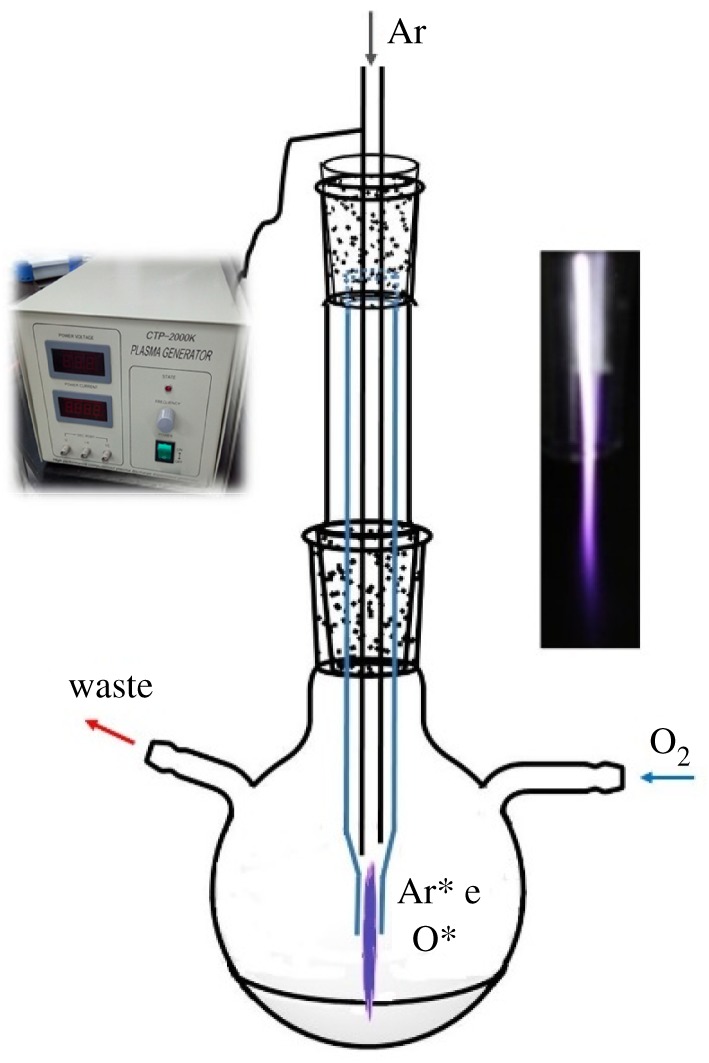
Schematic view of the experimental set-up.

The schematic view of the experiment set-up is shown in figure 1. It consisted of two coaxial glass tubes with a hollow steel needle in the centre of the inner tube. The hollow steel needle with a tip radius of about 100 µm served as a high-voltage (HV) electrode, which was directly connected to a pulsed direct current (DC) power supply, and was also used for carrying and guiding the working gas flow. There was a nozzle outlet at the end of the inner tube with an inner diameter of approximately 2 mm. The distance between the needle tip and the nozzle was about 5 mm. The glass tube was fixed into a round-bottom flask with a distance of about 3 mm between the nozzle and the solution surface. The working gas, argon (99.99%) passed through the hollow steel needle to generate the plasma jet, while O_2_ (99.9%) was introduced into the round-bottom flask, serving as the surrounding gas and oxygen source. Before turning on the power supply, Ar and O_2_ were allowed to flow into the flask for 5 min. Then, a high pulsed DC voltage was applied to the HV electrode, and a plasma jet was generated at the end of the needle inside the glass tube. The synthesis process was carried out by applying the plasma jet to the chlorobenzene solution of C_60_.

## 3. Results and discussion

### 3.1. Synthesis of C_60_O_n_

Initially, the applied discharge voltage of 3.5 kV was fixed, Ar and O_2_ flow rates were kept at 0.2 and 0.8 l min^−1^, respectively. Under the above APNPJ conditions, a solution of C_60_ (7.2 mg, 0.01 mmol) in 3 ml of chlorobenzene was treated at 10°C for 10 min to give a claret-red solution along with brown precipitates, which did not dissolve in commonly used organic solvents [14,16]. The solution was analysed by HPLC on a Buckyprep column (4.6 × 250 mm) (table 1, entry 1).

**Table 1.** Optimization of the plasma jet-promoted reaction of C_60_ with O_2_^a^. 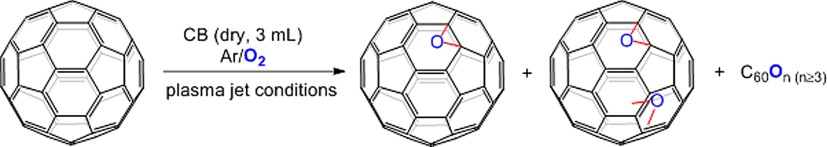

entry	Ar/O_2_ (l min^−1^)	V kV^−1^	reaction temp. (°C)	reaction time (min)	HPLC peak area for C_60_O^b^	HPLC peak area for C_60_O_2_^b^
1	0.2/0.8	3.5	10	10	7552	1528
2	0.2/0.8	3.5	0	10	9017	1953
3	0.2/0.8	3.5	−10	10	8915	1985
4	0.2/0.8	3.5	0	15	8586	2526
5	0.2/0.8	3.5	0	5	5924	658
6	0.2/0.8	4.0	0	10	5937	1408
7	0.2/0.8	3.0	0	10	6346	755
8	0.2/0.4	3.5	0	10	9273	2643
9	0.2/0.2	3.5	0	10	7949	2080
10	0.3/0.4	3.5	0	10	9023	2188
11	0.2/0.4	3.5	0	25	3190	3354
12	0.2/0.8	3.5	0	25	5205	3764
13	0.2/0.8	3.5	−10	25	5944	5362
14	0.2/0.8	3.5	−20	25	6718	6306
15	0.2/0.8	3.5	−30	25	2554	4008
16	0.2/0.8	3.5	−20	30	3104	4376
17	0.2/0.8	3.5	−20	20	6882	4157

^a^Unless otherwise noted, the reaction was performed using 7.2 mg of C_60_ in anhydrous chlorobenzene (3 ml) at the set temperature under the plasma conditions for the designated time.

^b^The HPLC peak area with an injection of 8 µl of the reaction mixture.

### 3.2. Structural assignments

**Figure 2.**
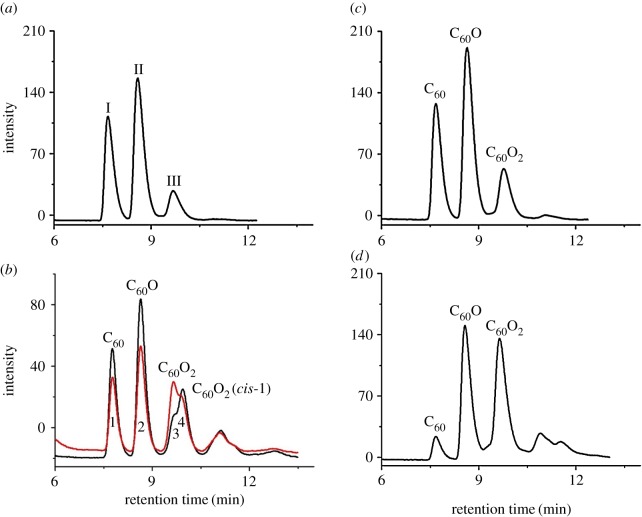
(*a*) HPLC trace for the reaction mixture treated by APNPJ under conditions in entry 1 of table 1. (*b*) Black line: HPLC trace for the reaction mixture of C_60_ and *m*-CPBA; red line: HPLC trace after addition of the isolated C_60_O_2_ under the APNPJ conditions to the reaction mixture of C_60_ and *m*-CPBA. (*c*) HPLC trace giving the highest yield of C_60_O under APNPJ conditions. (*d*) HPLC trace giving the highest yield of C_60_O_2_ under APNPJ conditions. The mobile phase on the Buckyprep (4.6 × 250 mm) column was toluene (1 ml min^−1^).

**Figure 3.**
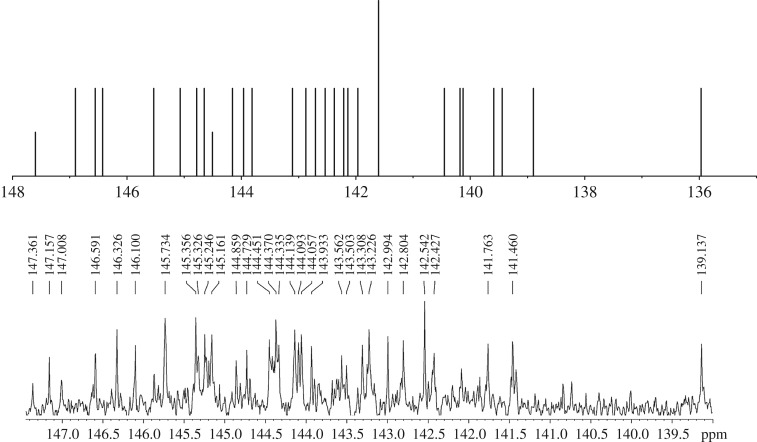
Comparison of the calculated ^13^C NMR spectrum of the *e* isomer of C_60_O_2_ with the experimental ^13^C NMR spectrum of peak **III**.

As shown in figure 2*a*, three peaks were observed for the reaction mixture. Peaks **I** and **II** could be conclusively assigned as unreacted C_60_ and the known epoxide C_60_O, respectively, by comparison of their retention times with those of the authentic samples. The structure of C_60_O was further unambiguously confirmed by comparison of its HR-MS (MALDI-TOF), ^13^C NMR, UV-vis and FT-IR spectra with those reported previously [12–14]. A series of work has been done to identify the possible structure for peak **III**. Interestingly, peak **III** had a different retention time from that of the *cis*-1 isomer of C_60_O_2_, instead it was eluted out at the same retention time as that of the unknown minor isomer of C_60_O_2_ produced by the oxidation of C_60_ with *m*-CPBA (figure 2*b*) [14]. The product contained in peak **III** was separated out by semi-preparative HPLC equipped with a Buckyprep column (10 × 250 mm) to allow structural analysis. The fraction for peak **III** was evaporated *in vacuo* and isolated as a brown powder, which had poor solubility in common good solvents including *o*-dichlorobenzene (ODCB) for fullerenes. The high-resolution mass spectrum HR-MS (MALDI-TOF) of the product showed a strong ion peak at 751.9878, which matched with the calculated molecular ion peak (751.9893, [M]^+^) of dioxide C_60_O_2_. The infrared spectrum for peak **III** in a KBr pellet revealed no bands above 1550 cm^−1^ and thus no C–H or C=O moiety was present. The peaks for the fullerene cage were observed at 1427, 1182, 563 and 525 cm^−1^. There were also other peaks due to the derivatization of the fullerene skeleton. The UV-vis spectrum of a given bisadduct of C_60_ depends mostly on the addition pattern rather than the nature of the addend. Hence, it is possible to use the UV-vis spectra as a diagnostic tool for the structural assignment of the newly synthesized bisadducts. The UV-vis spectrum for peak **III** exhibited a broad absorption band near 466 nm, and was almost the same as that of analogous *e* regioisomers of C_62_(COOEt)_4_ and C_60_(NCOOEt)_2_ [34–36]. The ^13^C NMR spectrum for peak **III** in CS_2_/CDCl_3_ with chromium(III) tris(acetylacetonate) as the relaxation reagent was obtained (figure 3, also see electronic supplementary material, figures S8 and S9). There were about 30 dominant peaks for the sp^2^ carbons of C_60_ in the ^13^C NMR spectrum, suggesting that peak **III** predominantly consisted of one C_60_O_2_ isomer. The attempt to separate the possible isomers from each other in peak **III** by the recycling preparative HPLC on a Buckyprep or Buckyprep-M column failed (see electronic supplementary material, figures S13 and S14).

In order to gain further insight into the molecular structure for peak **III**, theoretical calculations of the ^13^C NMR chemical shifts based on gauge-including atomic orbitals (GIAO) [37] for eight isomers of C_60_O_2_ were performed at the B3LYP/6-311+G (2df, 2pd) level with the Gaussian 09 program [38] and compared (see figure 3 and electronic supplementary material, figure S15). The linear correlation between the calculated ^13^C NMR chemical shifts and experimentally obtained chemical shifts of *cis*-1 isomer of C_60_O_2_ was very good (*R* = 0.999, see electronic supplementary material, figure S16), indicating that the calculated ^13^C NMR chemical shifts based on GIAO were reliable. The comparison of the number and relative position of signals between computational and experimental ^13^C NMR spectra suggested that only the calculated ^13^C NMR spectrum of the *e* isomer could match with that of peak **III** well, consistent with the conclusion from the above-mentioned UV-vis spectrum analysis.

### 3.3. Optimization of reaction conditions

With the preliminary result (table 1, entry 1) in hand, the reaction conditions were optimized by changing the reaction time, reaction temperature, discharge voltage and gas flow rate to obtain the highest yield of C_60_O, and the results are summarized in table 1. The reaction temperatures and reaction times were varied, and it was found that 0°C and 10 min were optimal (table 1, entries 2–5). The discharge voltage was also examined, increasing or decreasing the voltage did not improve the yield (table 1, entries 6 and 7). Next, the gas flow rate was further investigated. A combination of 0.2 and 0.4 l min^−1^ for Ar and O_2_ resulted in the highest yield of C_60_O (table 1, entry 8). However, further decreasing the O_2_ flow rate was not beneficial to the reaction efficiency (table 1, entry 9). When the flow rate of the working gas was increased to 0.3 l min^−1^, a slightly decrease in product yield was observed (table 1, entry 10). The plasma plume was too weak to initiate the reaction when the Ar flow rate was 0.1 l min^−1^. Therefore, the optimal reaction conditions for the synthesis of C_60_O were determined as follows: the applied discharge voltage of 3.5 kV, the Ar flow rate of 0.2 l min^−1^, the O_2_ flow rate of 0.4 l min^−1^, the reaction temperature of 0°C and the reaction time of 10 min. In order to achieve a higher yield of C_60_O_2_, the reaction time was prolonged from 10 to 25 min; a slightly higher yield was obtained (table 1, entry 11). Increasing the O_2_ flow rate from 0.4 l min^−1^ to 0.8 l min^−1^ further improved the yield of C_60_O_2_ (table 1, entry 12). It was found that decreasing the reaction temperature to −20°C afforded the highest yield of C_60_O_2_ (table 1, entries 13−15). Further increasing or decreasing the reaction time was not beneficial to the formation of C_60_O_2_ (table 1, entries 16 and 17). As a result, the optimal reaction conditions for C_60_O_2_ were achieved by lowering the temperature to −20°C and prolonging the reaction time to 25 min as well as increasing the O_2_ flow rate to 0.8 l min^−1^ (table 1, entry 14). Under the optimum reaction conditions, the isolated yields for C_60_O and C_60_O_2_ were 44% (figure 2*c*) and 21% (figure 2*d*), respectively. Notably, this method could be successfully applied to a scale-up reaction. The reaction of C_60_ (36 mg, 0.05 mmol) under the optimal conditions for the synthesis of C_60_O could afford 37% yield after prolonging the reaction time to 45 min. Likewise, treatment of C_60_ (36 mg, 0.05 mmol) with the plasma jet at −20°C for 90 min provided C_60_O_2_ in 11% yield. In addition, we had also preliminarily studied the oxidation of C_70_ under the plasma conditions. Unfortunately, much less of C_70_ was converted when compared with C_60_, probably due to its lower reactivity (see electronic supplementary material, figures S17−S19). Therefore, the oxidation of C_70_ by plasma jet was not investigated in more details.

### 3.4. Optical emission spectra and reaction mechanism

**Figure 4.**
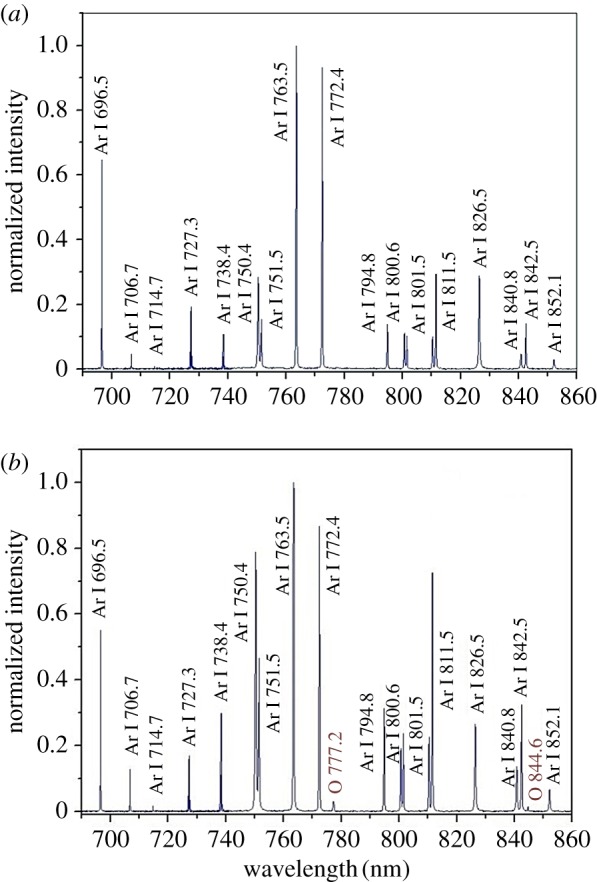
Optical emission spectra in the range of 650–860 nm: (*a*) Ar, (*b*) the mixture of Ar and O_2_.

To shed light on the mechanism of the plasma-promoted formation of C_60_O_n_, optical emission spectroscopy was employed to identify the reactive species generated by the Ar/O_2_ APNPJ. Figure 4 shows the comparison of the optical emission spectra of both Ar and Ar/O_2_ plasma jets in the range of 650−860 nm. Two additional emission peaks, which were assigned to the reactive atomic oxygen species O* (777.2 nm, 3*p*^5^*P*–3*s*^5^*S*) and O* (844.6 nm, 3*p*^3^*P*–3*s*^3^*S*), were detected in the Ar/O_2_ APNPJ [39–42]. The generation of the reactive oxygen species as well as the production of C_60_O_n_ are shown in equations (3.1)−(3.5) [43–46]. Firstly, the plasma jet initiated the reaction and produced active species including activated Ar*, electrons and so on. Then, these active species collided with dioxygen to produce the active atomic oxygen species (equations (3.1)−(3.4)), which reacted with C_60_ in solution to produce C_60_O and C_60_O_2_.

Different from the dominant formation of the *cis*-1 isomer of C_60_O_2_ by the oxidation with *m*-chloroperoxybenzoic acid, the *e* isomer of C_60_O_2_ was selectively generated under our plasma jet conditions, indicating that their reaction mechanisms should be different. Previous theoretical study showed that the *cis*-1 isomer was the most stable and the *e* isomer was the second most stable thermodynamically among the eight isomers of C_60_O_2_ [47]. Therefore, the selective formation of the *e* isomer of C_60_O_2_ under our plasma jet conditions should be governed by a kinetic process, most probably due to very reactive oxygen atom species.

3.1 $$\begin{array}{rl} & \text{e}+{\text{O}}_{2}\to {\text{O}}^{*}+\text{O}+\text{e}, \end{array}$$

3.2 $$\begin{array}{rl} & \text{e}+\text{O}\to {\text{O}}^{*}+\text{e}, \end{array}$$

3.3 $$\begin{array}{rl} & {\text{Ar}}^{*}+{\text{O}}_{2}\to {\text{O}}^{*}+\text{O}+\text{Ar}, \end{array}$$

3.4 $$\begin{array}{rl} & {\text{Ar}}^{*}+\text{O}\to {\text{O}}^{*}+\text{Ar} \end{array}$$

3.5 $$\begin{array}{rl} \text{and}\;\; & {\text{nO}}^{*}+{\text{C}}_{60}\to {\text{C}}_{60}{\text{O}}_{n}. \end{array}$$

## 4. Conclusion

In summary, we have successfully developed the plasma jet-promoted synthesis of fullerene oxides. Under the respectively optimized conditions, C_60_O and C_60_O_2_ were isolated in 44% and 21% yields, which are the highest reported so far. More importantly, the generated C_60_O_2_ under the plasma jet conditions dominantly consisted of the *e* isomer, which exhibited very different regioselectivity compared to the *cis*-1 isomer as the major product of C_60_O_2_ formed from the oxidation of C_60_ with *m*-chloroperoxybenzoic acid. This is the first time that plasma jet is applied to fullerene chemistry, and this work may be of interest to synthetic chemists for developing the unique plasma technique to promote chemical reactions.

## 5. General procedure and characterization data

Synthesis of C_60_O as the major product: under the applied discharge voltage of 3.5 kV, the Ar flow rate of 0.2 l min^−1^, the O_2_ flow rate of 0.4 l min^−1^, the chlorobenzene solution (3 ml) of C_60_ (0.01 mmol, 7.2 mg) was treated with the plasma jet at 0°C for 10 min, and colour of the solution turned from purple to claret-red. The resulting solution from five runs was combined and filtrated through a silica gel plug with carbon disulfide as the eluent to remove any insoluble material and then evaporated *in vacuo*. The residue was dissolved in toluene and separated by recycling preparative HPLC on a Buckyprep column (10 × 250 mm) with a mixture of toluene and *n*-hexane (1 : 1 v/v) as the eluent, giving unreacted C_60_ (10.9 mg, 31%), C_60_O (15.9 mg, 44%) and C_60_O_2_ (4.0 mg, 11%).

A scale-up reaction of C_60_O as the major product: under the applied discharge voltage of 3.5 kV, the Ar flow rate of 0.2 l min^−1^, the O_2_ flow rate of 0.4 l min^−1^, the chlorobenzene solution (15 ml) of C_60_ (0.05 mmol, 35.8 mg) was treated with the plasma jet at 0°C for 45 min, and colour of the solution turned from purple to claret-red. The resulting solution was filtrated through a silica gel plug with carbon disulfide as the eluent to remove any insoluble material and then evaporated *in vacuo*. The residue was dissolved in toluene and separated by recycling preparative HPLC on a Buckyprep column (10 × 250 mm) with a mixture of toluene and *n*-hexane (1 : 1 v/v) as the eluent, giving unreacted C_60_ (15.4 mg, 43%), C_60_O (13.5 mg, 37%) and C_60_O_2_ (1.8 mg, 5%).

^13^C NMR (100 MHz, CDCl_2_CDCl_2_ with chromium(III) tris(acetylacetonate) as a relaxation reagent all 4C unless indicated) *δ* 145.17 (8C), 145.06, 144.94 (2C), 144.23, 143.96, 143.85, 143.74, 143.40 (2C), 142.96, 142.95, 142.43 (2C), 142.25, 142.05, 141.83, 140.78, 89.53 (2C, sp^3^-*C* of C_60_); FT-IR *ν*/cm^−1^ (KBr) 1537, 1504, 1458, 1427, 1378, 1310, 1243, 1183, 1080, 876, 769, 742, 674, 625, 598, 568, 526, 498, 435; UV-vis (toluene) *λ*_max_/nm (log *ε*) 424 (3.19), 496 (3.07); UV-vis (CHCl_3_) *λ*_max_/nm (log *ε*) 255 (5.13), 323 (4.57), 412 (3.47), 420 (3.35), 494 (3.26); HR-MS (MALDI-TOF) *m/z* calcd for C_60_O [M]^+^ 735.9944, found 735.9909.

Synthesis of C_60_O_2_ as the major product: under the applied discharge voltage of 3.5 kV, the Ar flow rate of 0.2 l min^−1^, the O_2_ flow rate of 0.8 l min^−1^, the chlorobenzene solution (3 ml) of C_60_ (0.01 mmol, 7.2 mg) was treated with the plasma jet at −20°C for 25 min, and colour of the solution turned from purple to claret-red. The resulting solution from five runs was combined and filtrated through a silica gel plug with carbon disulfide as the eluent to remove any insoluble material and then evaporated *in vacuo*. The residue was dissolved in toluene and separated by recycling preparative HPLC on a Buckyprep column (10 × 250 mm) with a mixture of toluene and *n*-hexane (1 : 1 v/v) as the eluent, affording C_60_ (4.5 mg, 13%), C_60_O (11.6 mg, 32%) and C_60_O_2_ (7.8 mg, 21%).

A scale-up reaction of C_60_O_2_ isomers as the major product: under the applied discharge voltage of 3.5 kV, the Ar flow rate of 0.2 l min^−1^, the O_2_ flow rate of 0.8 l min^−1^, the chlorobenzene solution (15 ml) of C_60_ (0.05 mmol, 36.5 mg) was treated with the plasma jet at −20°C for 90 min, and colour of the solution turned from purple to claret-red. The resulting solution was filtrated through a silica gel plug with carbon disulfide as the eluent to remove any insoluble material and then evaporated *in vacuo*. The residue was dissolved in toluene and separated by recycling preparative HPLC on a Buckyprep column (10 × 250 mm) with a mixture of toluene and *n*-hexane (1 : 1 v/v) as the eluent, affording unreacted C_60_ (7.4 mg, 20%), C_60_O (10.6 mg, 28%) and C_60_O_2_ (4.0 mg, 11%).

^13^C NMR (100 MHz, CS_2_/CDCl_3_ with chromium(III) tris(acetylacetonate) as a relaxation reagent) *δ* 147.36, 147.16, 147.01, 146.59, 146.33, 146.10, 145.73, 145.36, 145.33, 145.25, 145.16, 144.86, 144.73, 144.45, 144.37, 144.34, 144.14, 144.09, 144.06, 143.93, 143.56, 143.50, 143.31, 143.23, 142.99, 142.80, 142.54, 142.43, 141.76, 141.46, 139.14, 90.96 (sp^3^-*C* of C_60_), 89.84 (sp^3^-*C* of C_60_), 89.26 (sp^3^-*C* of C_60_); FT-IR *ν*/cm^−1^ (KBr) 1539, 1507, 1456, 1427, 1376, 1305, 1240, 1182, 1085, 1036, 964, 796, 770, 746, 625, 563, 525, 497, 432; UV-vis (toluene) *λ*_max_/nm (log *ε*) 466 (3.73); UV-vis (CHCl_3_) *λ*_max_/nm (log *ε*) 250 (5.12), 301 (4.72), 466 (3.63); HR-MS (MALDI-TOF) *m/z* calcd for C_60_O_2_ [M]^+^ 751.9893, found 751.9878.

## Supplementary Material

Electronic supplementary material for the synthesis of [60]fullerene oxides by plasma jet

## Data accessibility

The datasets supporting this article have been uploaded as part of the electronic supplementary material.
