# Supplementary material for: Highly efficient synthesis of [60]fullerene oxides by plasma jet
Source: R Soc Open Sci. 2017 Sep 6;4(9):170658. doi: 10.1098/rsos.170658 (PMC5627109; doi:10.1098/rsos.170658)
Supplement: Electronic supplementary material for the synthesis of [60]fullerene oxides by plasma jet [file rsos170658supp1.pdf]

Electronic Supplementary Material

Highly efficient synthesis of [60]fullerene oxides by plasma jet

Sheng-Peng Jiang[1], Shengxia Duan[2], Kai-Qing Liu[1], Xiao-Yu Yang[1], Cheng Cheng[2], Jiaxing Li[2,3]*, Guan-Wu Wang[1]*

[1]CAS Key Laboratory of Soft Matter Chemistry, Collaborative Innovation Center of Chemistry for Energy Materials, Hefei National Laboratory for Physical Sciences at Microscale and Department of Chemistry, University of Science and Technology of China, Hefei, Anhui 230026, P. R. China

[2]Institute of Plasma Physics, Chinese Academy of Sciences, P.O. Box 1126, Hefei, 230031, P.R. China

[3]Collaborative Innovation Center of Radiation Medicine of Jiangsu Higher Education Institutions, P.R. China

Authors for correspondence:

Guan-Wu Wang; Jiaxing Li

e-mail: gwang@ustc.edu.cn; lijx@ipp.ac.cn

Table of contents

Schematic view of the experimental setup S3

HR-MS (MALDI-TOF), ^{13}C NMR, UV-vis and IR spectra of $C_{60}O$ S4–6

HR-MS (MALDI-TOF), ^{13}C NMR, UV-vis and IR spectra of $C_{60}O_2$ S7–9

Recycling separation of $C_{60}O_2$ S10

Comparison between calculated ^{13}C NMR spectra of $C_{60}O_2$ isomers and experimental ^{13}C NMR spectrum for peak **III** S11

Linear correlation between experimental and GIAO (B3LYP/6-311+ G (2df, 2pd)) calculated ^{13}C NMR spectra for *cis*-1 isomer of $C_{60}O_2$ S12

Oxidation of C_{70} under the plasma conditions S12–13

The xyz coordinates for the structure of eight isomers of $C_{60}O_2$ S14–37

Reference S37

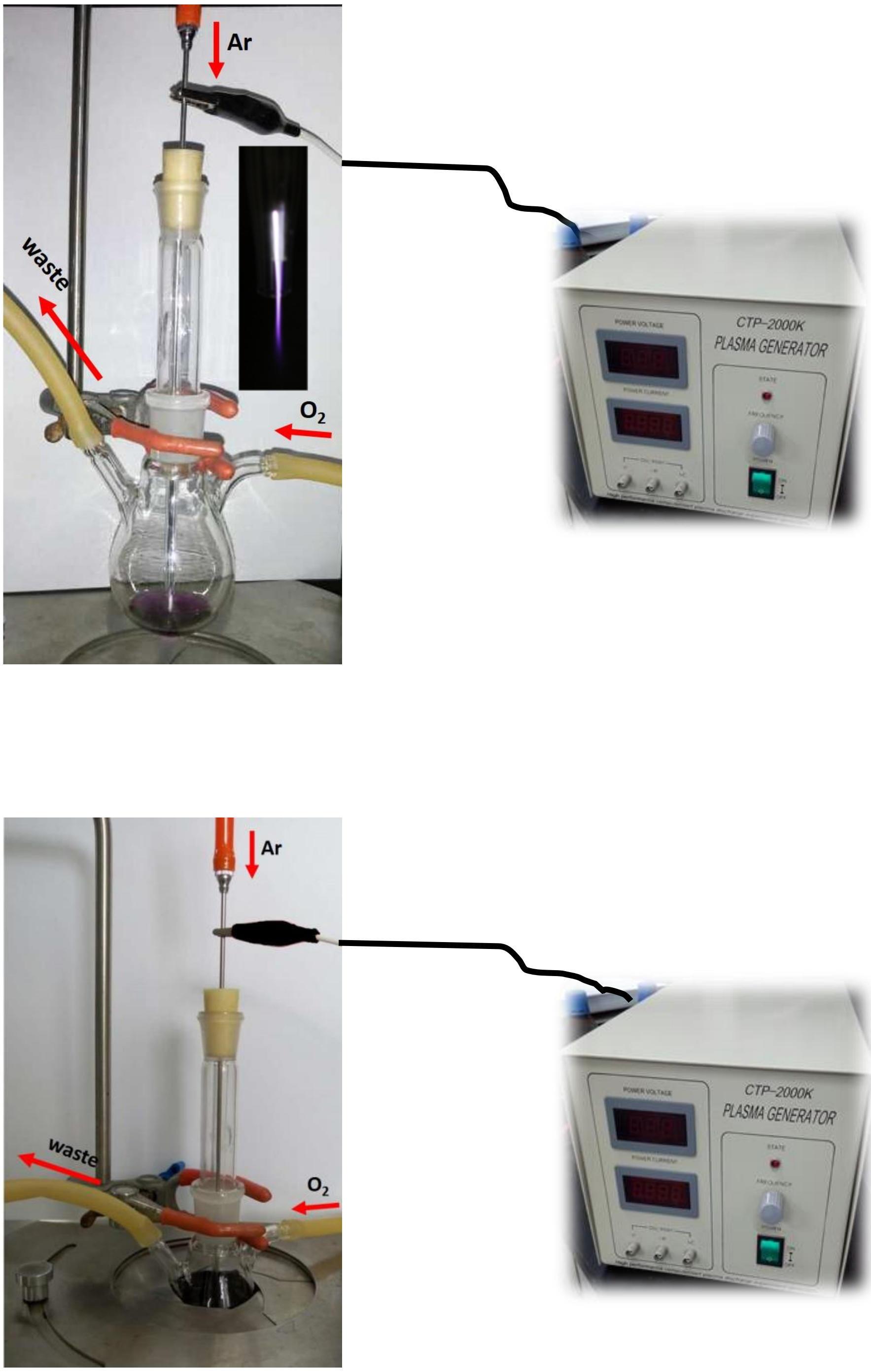

Figure S1. Schematic view of the experimental setup

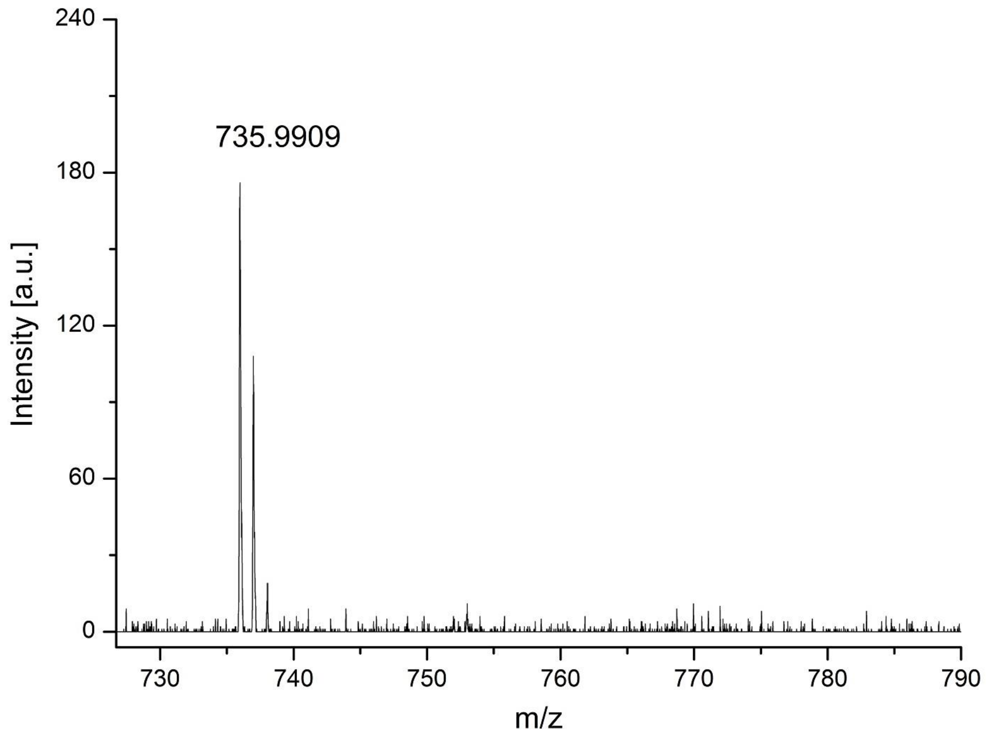

Figure S2. The HR-MS (MALDI-TOF) spectrum of $C_{60}O$

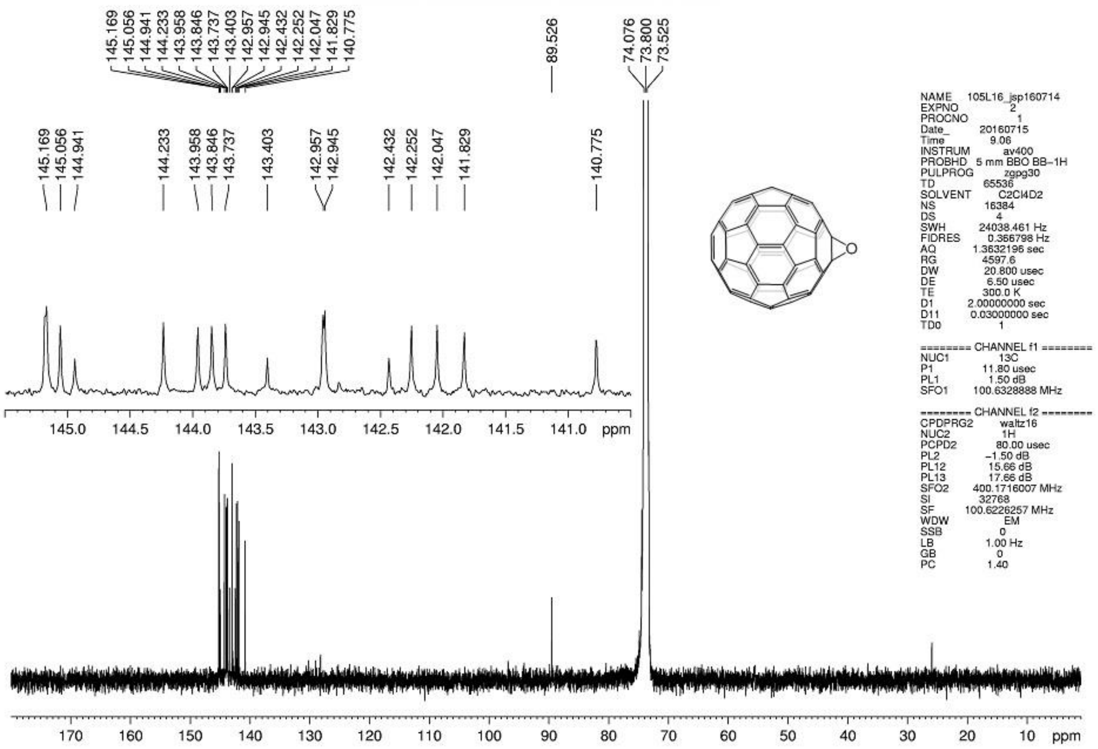

Figure S3. The ^{13}C NMR (100 MHz, $C_2D_2Cl_4$ with $Cr(acac)_3$ as a relaxation reagent)

spectrum of $C_{60}O$

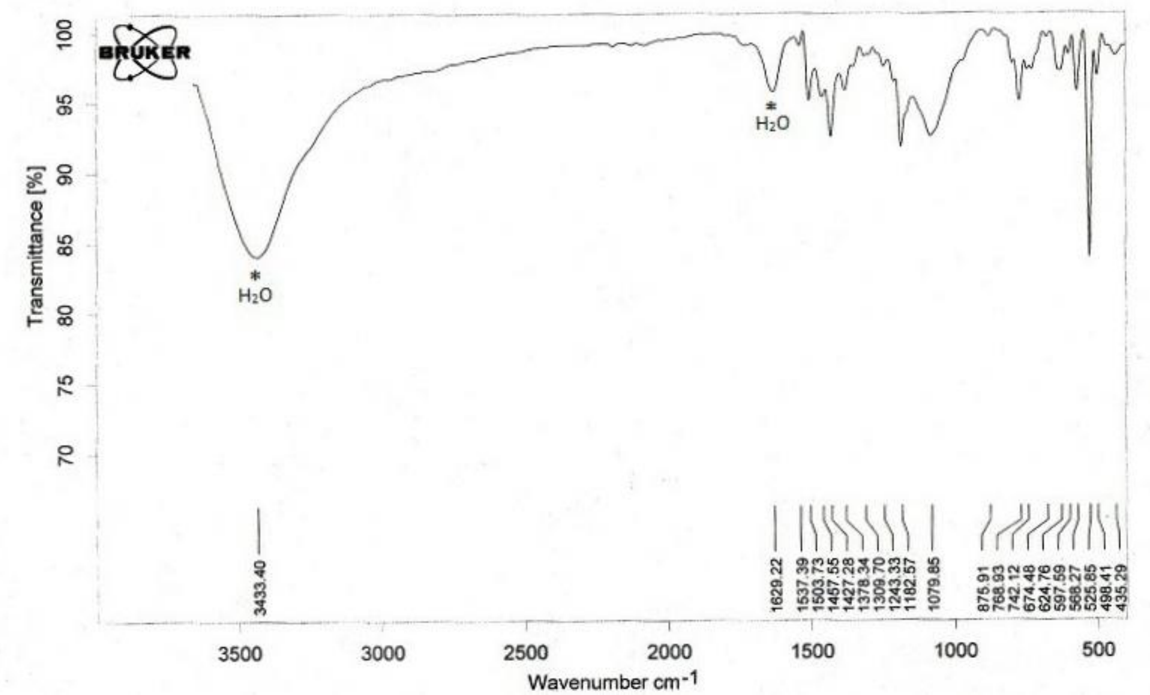

Figure S4. The IR spectrum of $C_{60}O$

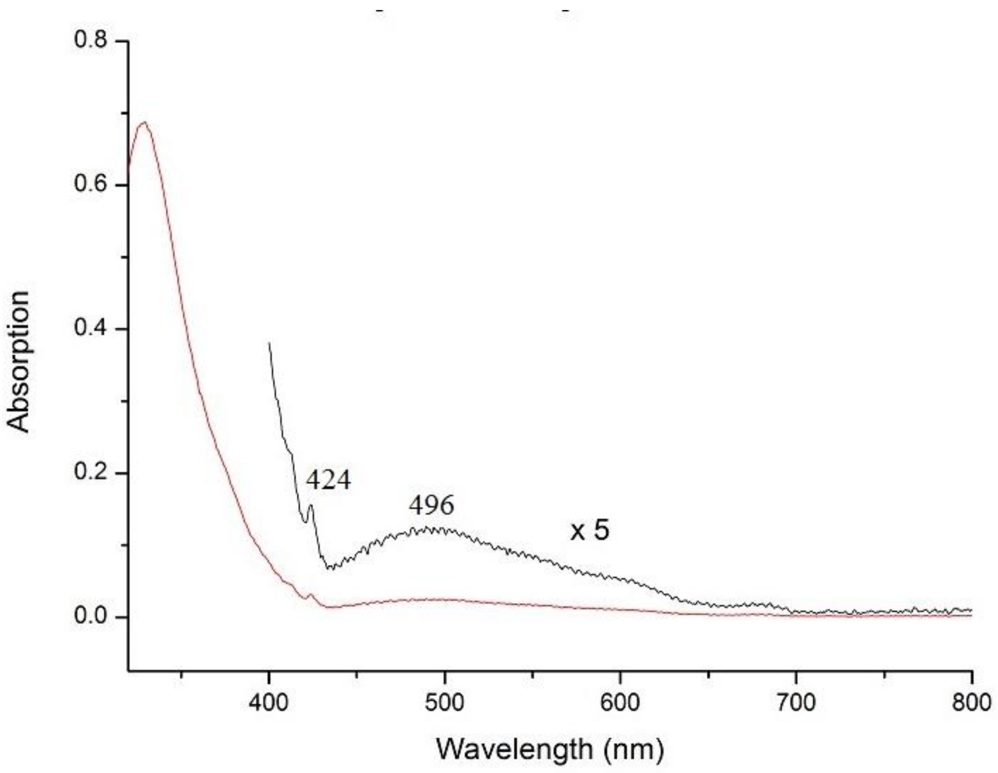

Figure S5. The UV-vis spectrum of $C_{60}O$ in toluene

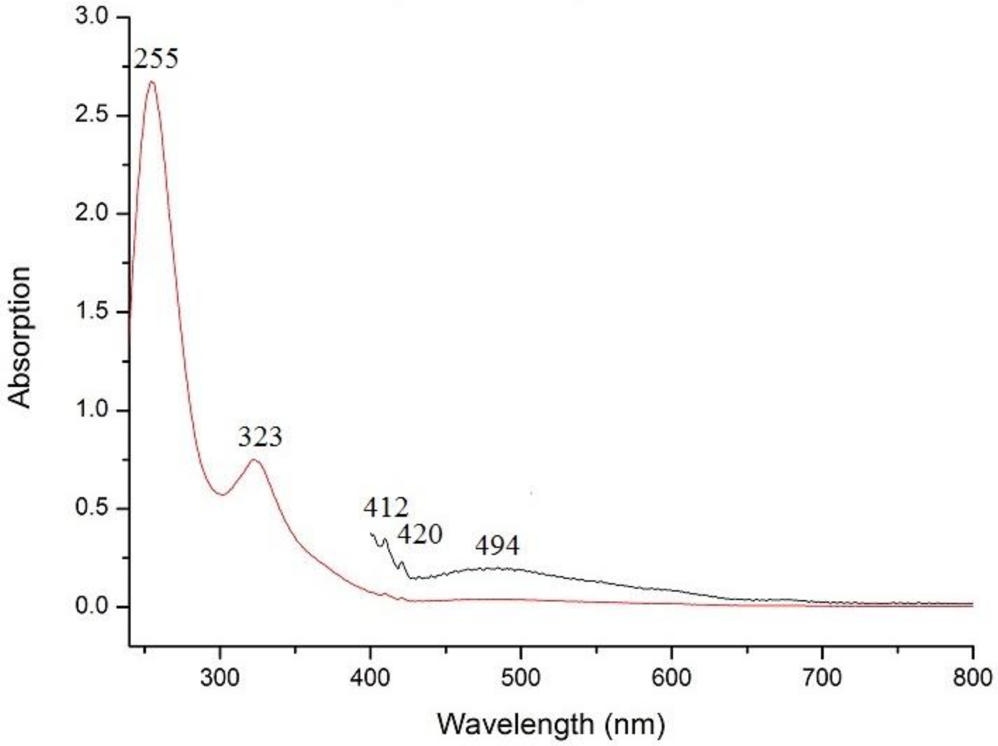

Figure S6. The UV-vis spectrum of $C_{60}O$ in $CHCl_3$

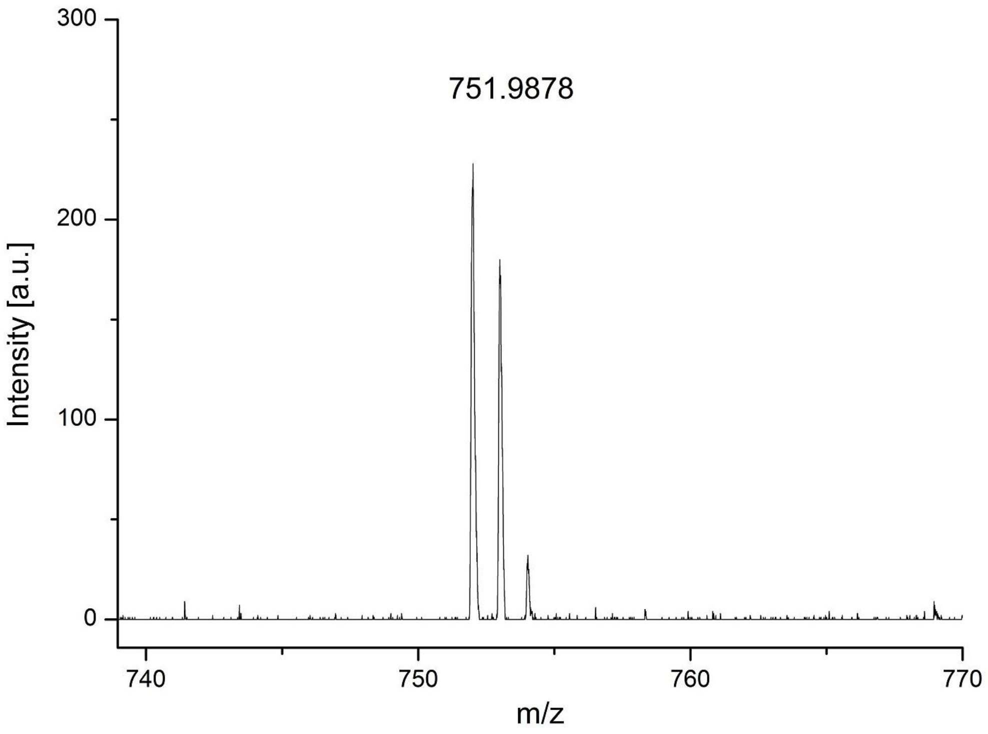

Figure S7. The HR-MS (MALDI-TOF) spectrum of $C_{60}O_2$

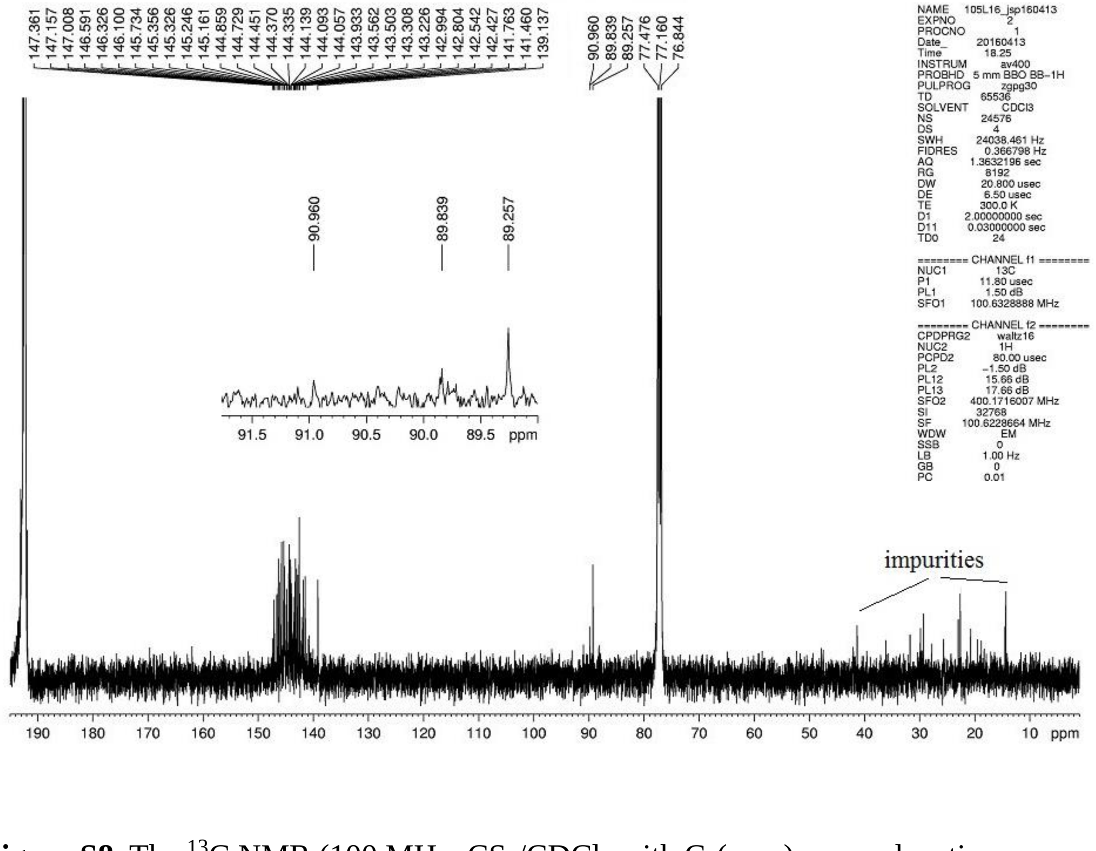

Figure S8. The ^{13}C NMR (100 MHz, $CS_2/CDCl_3$ with $Cr(acac)_3$ as a relaxation reagent) spectrum of $C_{60}O_2$

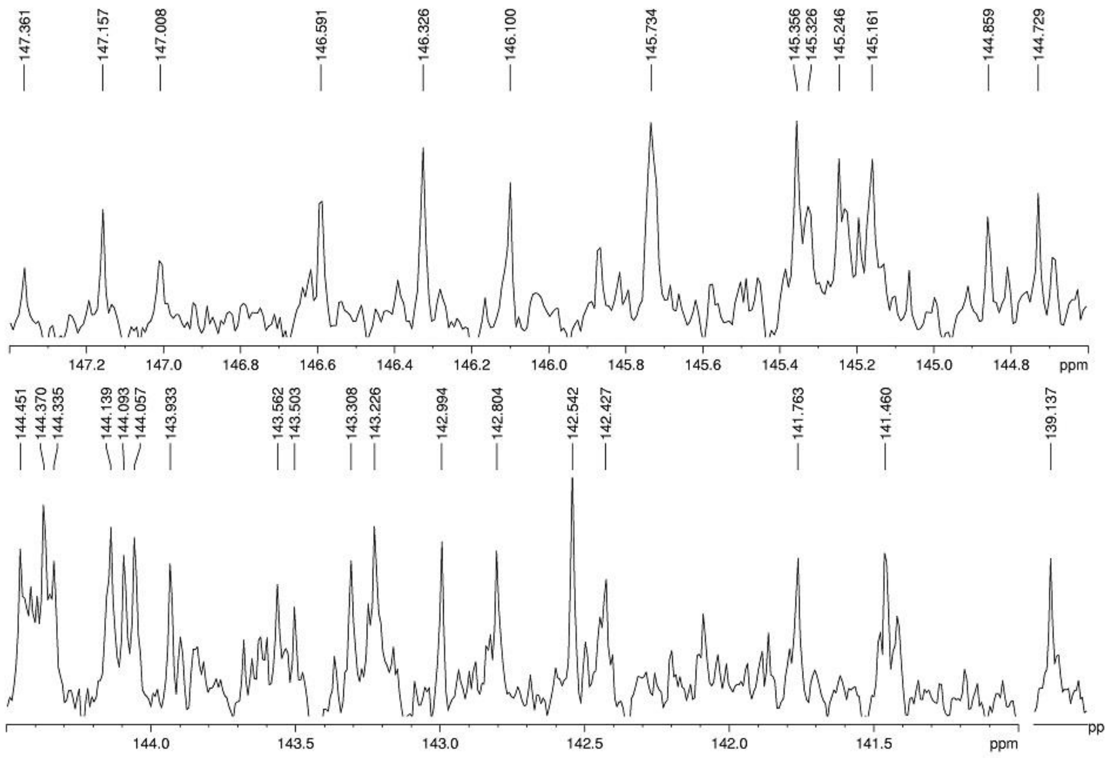

Figure S9. The expanded ^{13}C NMR (100 MHz, $CS_2/CDCl_3$ with $Cr(acac)_3$ as a relaxation reagent) spectrum of $C_{60}O_2$

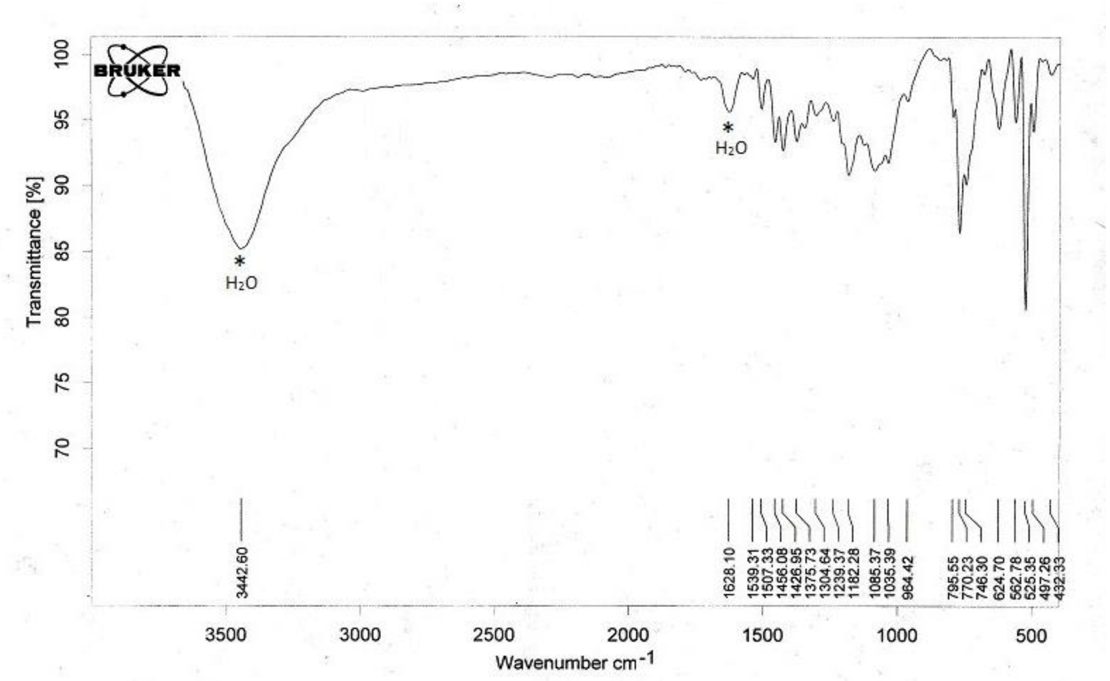

Figure S10. The IR spectrum of $C_{60}O_2$

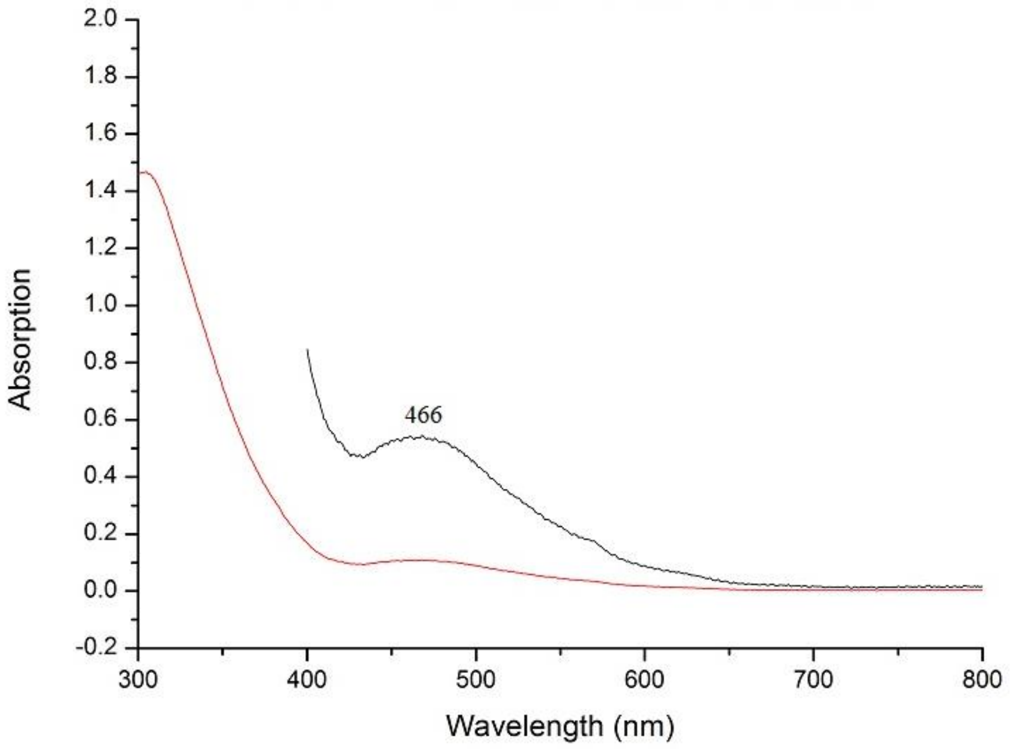

Figure S11. The UV-vis spectrum of $C_{60}O_2$ in toluene

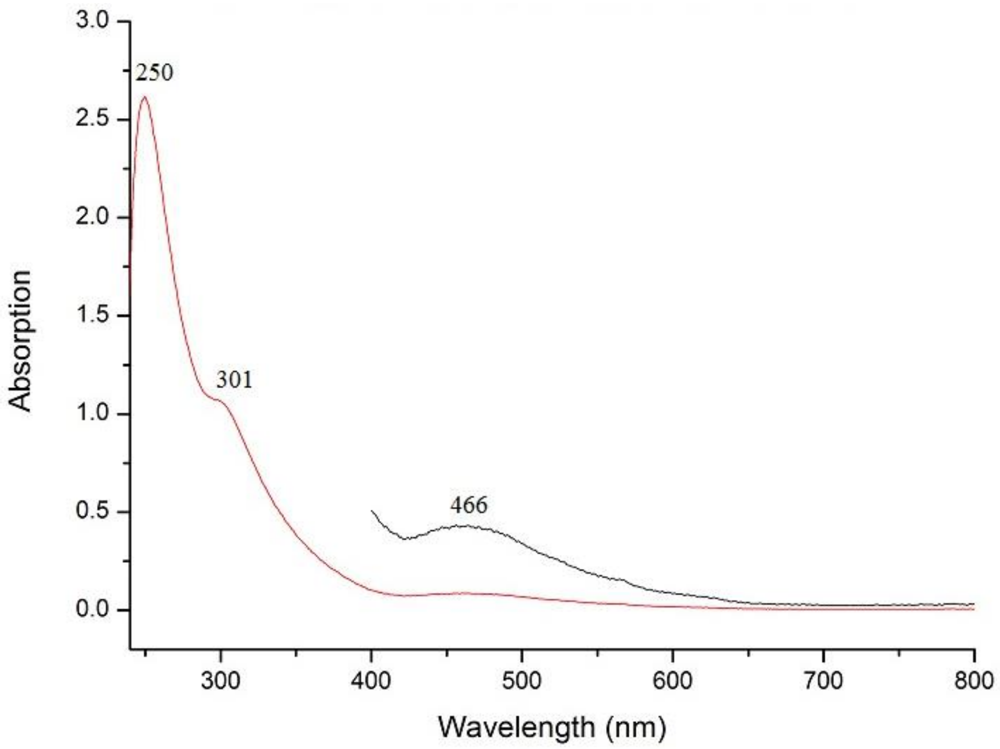

Figure S12. The UV-vis spectrum of $C_{60}O_2$ in $CHCl_3$

Recycling separation of $C_{60}O_2$

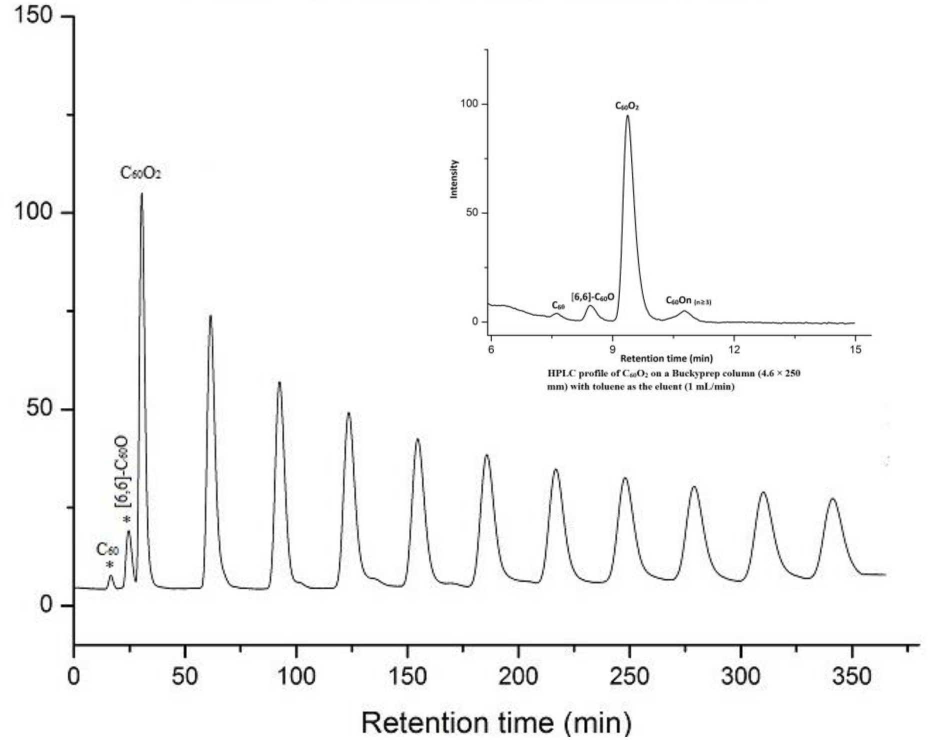

Figure S13. Recycling HPLC profile of $C_{60}O_2$ on a Buckyprep column (10 × 250 mm) with toluene as the eluent (5 mL/min)

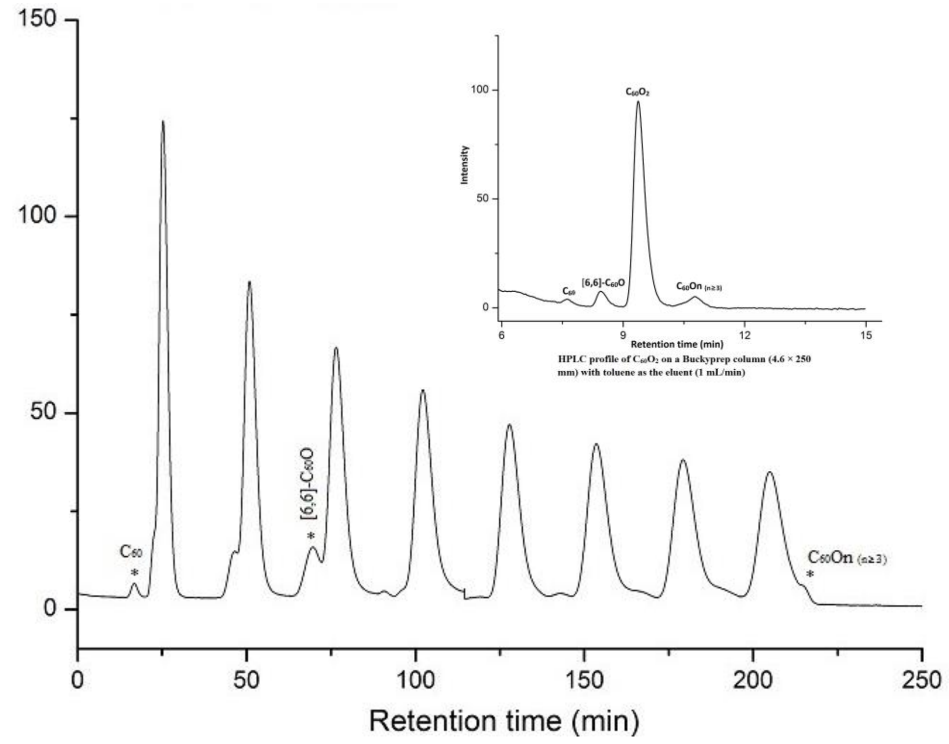

Figure S14. Recycling HPLC profile of $C_{60}O_2$ on a Buckyprep-M column (10 × 250 mm) with toluene as the eluent (5 mL/min)

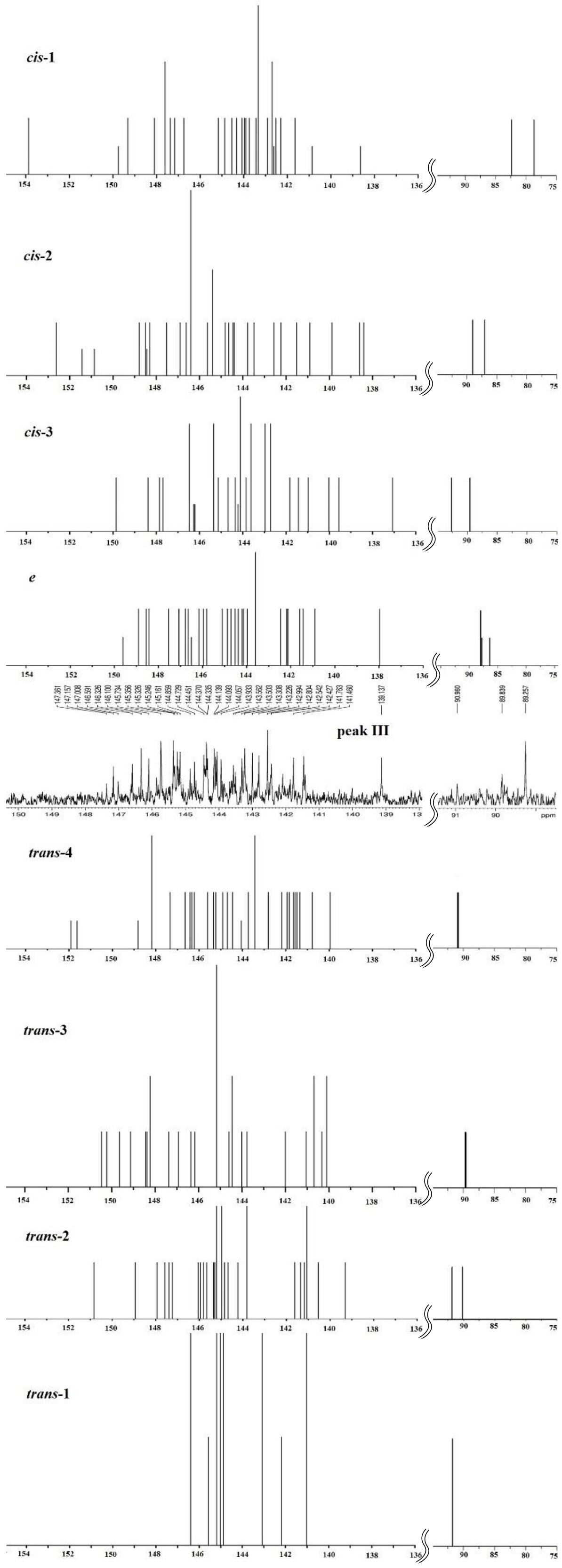

Figure S15. Comparison between calculated ^{13}C NMR spectra of $C_{60}O_2$ isomers and experimental ^{13}C NMR spectrum for peak **III**

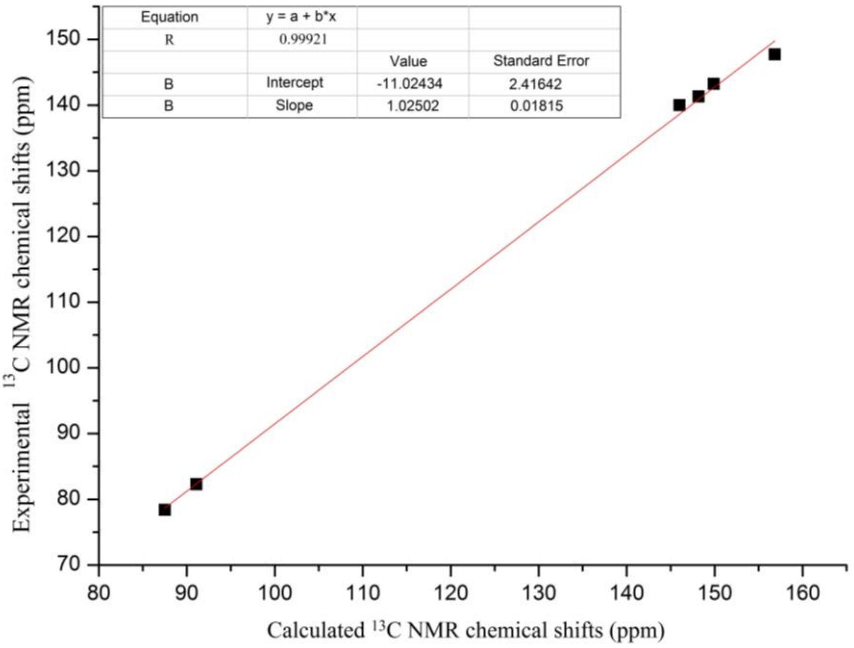

Figure S16. Linear correlation between experimental[1] and GIAO (B3LYP/6-311+ G (2df, 2pd)) calculated ^{13}C NMR data for *cis*-1 isomer of $C_{60}O_2$: $\delta_{(exp)} = 1.02502\delta_{(cal)}-11.02434$ (R = 0.99921)

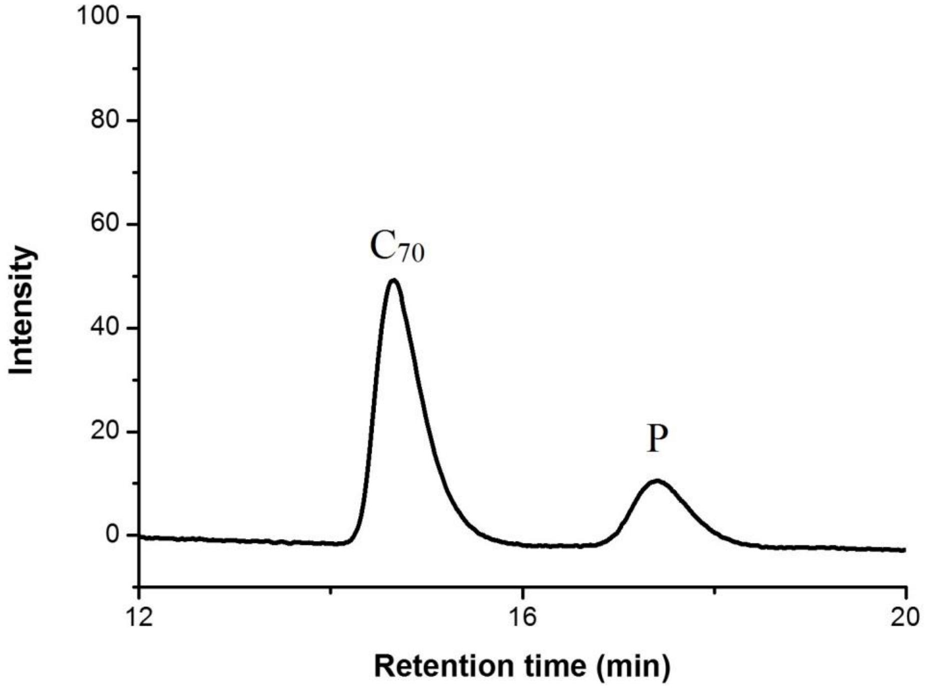

Figure S17. HPLC profile of C_{70} treated with plasma jet (Ar: 200 mL/min, O_2: 400 mL/min, 3.5 kV, 0 ^{o}C, 15 min) on a Buckyprep column (4.6 × 250 mm) with toluene as the eluent (1 mL/min)

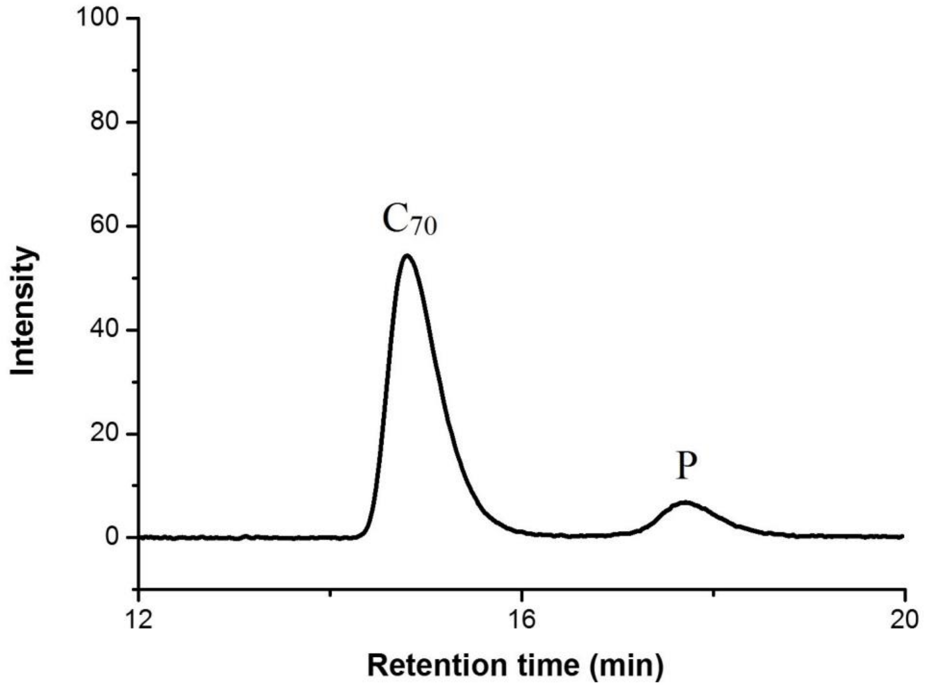

Figure S18. HPLC profile of C_{70} treated with plasma jet (Ar: 200 mL/min, O_2: 400 mL/min, 3.5 kV, 0 °C, 30 min) on a Buckyprep column (4.6 × 250 mm) with toluene as the eluent (1 mL/min)

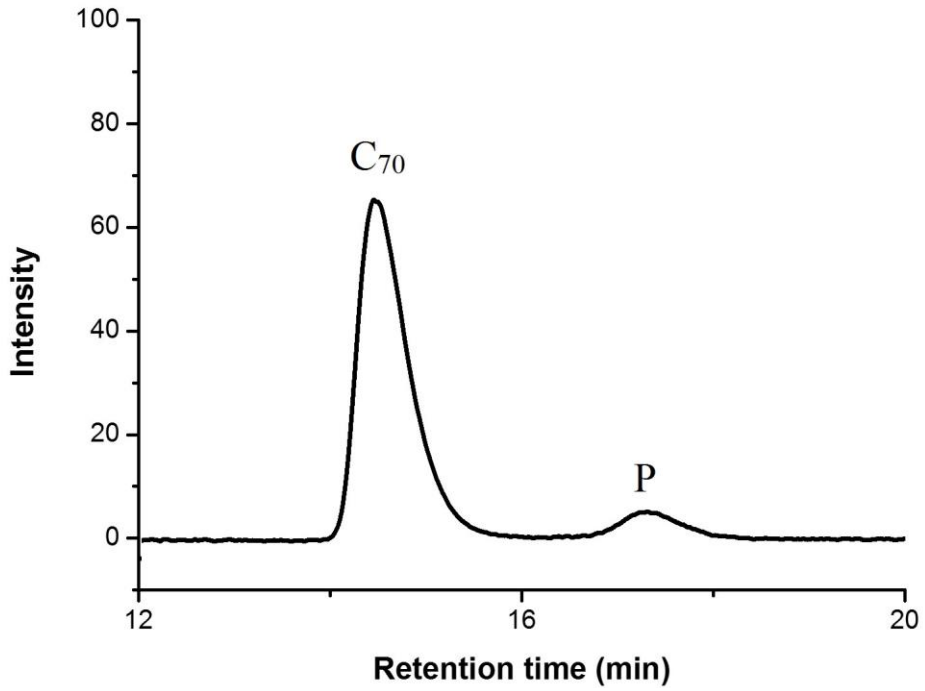

Figure S19. HPLC profile of C_{70} treated with plasma jet (Ar: 200 mL/min, O_2: 400 mL/min, 3.5 kV, –10 °C, 15 min) on a Buckyprep column (4.6 × 250 mm) with toluene as the eluent (1 mL/min)

The xyz coordinates for the structure of $C_{60}O_2$ (*cis*-1)

Charge = 0 Multiplicity = 1

C	2.58423	-0.99588	-2.30013
C	1.82872	0.01545	-3.02217
C	1.83007	1.34066	-2.57934
C	2.60067	1.7169	-1.40341
C	3.33269	0.74146	-0.71865
C	3.32216	-0.63958	-1.17228
C	1.80606	-2.21966	-2.304
C	0.57238	-1.96244	-3.03201
C	0.59279	-0.57984	-3.47575
C	-0.58283	0.17831	-3.47251
C	0.63169	2.13689	-2.62453
C	1.89752	2.75523	-0.69128
C	1.89752	2.75523	0.69128
C	2.60067	1.7169	1.40341
C	3.33269	0.74146	0.71865
C	3.32216	-0.63958	1.17228
C	3.31454	-1.49469	0.00000
C	2.56312	-2.67262	0.00000
C	1.79317	-3.04086	-1.17453
C	-0.62272	-2.53724	-2.59901

C	-0.63617	-3.39134	-1.42485
C	0.54688	-3.63811	-0.72613
C	0.54688	-3.63811	0.72613
C	1.79317	-3.04086	1.17453
C	1.80606	-2.21966	2.304
C	2.58423	-0.99588	2.30013
C	1.82872	0.01545	3.02217
C	1.83007	1.34066	2.57934
C	0.68406	3.11367	-1.49803
C	-1.8129	1.81068	2.29476
C	-1.82292	2.63503	1.18002
C	-2.54441	2.23031	0.00000
C	-3.31273	1.05802	0.00000
C	-3.32883	0.21604	1.17129
C	-1.82856	-0.42488	3.02101
C	-0.58283	0.17831	3.47251
C	-0.56443	1.55942	3.03324
C	0.63169	2.13689	2.62453
C	0.68406	3.11367	1.49803
C	-0.62051	3.4005	0.75671
C	-1.82292	2.63503	-1.18002
C	-1.8129	1.81068	-2.29476

C	-2.57968	0.58269	-2.29745
C	-3.32883	0.21604	-1.17129
C	-3.3498	-1.16606	-0.72878
C	-3.3498	-1.16606	0.72878
C	-2.62247	-2.1311	1.42264
C	-1.84949	-1.75306	2.59584
C	0.59279	-0.57984	3.47575
C	0.57238	-1.96244	3.03201
C	-0.62272	-2.53724	2.59901
C	-0.63617	-3.39134	1.42485
C	-1.87142	-3.13835	0.6979
C	-1.87142	-3.13835	-0.6979
C	-2.62247	-2.1311	-1.42264
C	-1.84949	-1.75306	-2.59584
C	-1.82856	-0.42488	-3.02101
C	-0.62051	3.4005	-0.75671
C	-0.56443	1.55942	-3.03324
C	-2.57968	0.58269	2.29745
O	0.04453	4.39121	1.51955
O	0.04453	4.39121	-1.51955

The xyz coordinates for the structure of $C_{60}O_2$ (*cis*-2)

Charge = 0 Multiplicity = 1

C	1.33859	2.6236	1.86904
C	2.02815	1.42346	2.37812
C	2.92215	0.71537	1.58223
C	3.3054	1.19881	0.22627
C	2.58949	2.44843	-0.30349
C	1.56823	3.08321	0.58117
C	-0.0086	2.60775	2.40138
C	-0.154	1.42605	3.23522
C	1.10556	0.7013	3.21479
C	1.10556	-0.7013	3.21479
C	2.92215	-0.71537	1.58223
C	3.37173	0.00000	-0.6514
C	2.81304	0.00000	-1.90145
C	2.11129	1.18885	-2.42171
C	1.97226	2.33862	-1.65417
C	0.70345	3.01701	-1.60872
C	0.45692	3.47261	-0.24767
C	-0.84604	3.47917	0.2593
C	-1.08303	3.03652	1.61442
C	-1.35991	0.72801	3.25441
C	-2.47472	1.17581	2.43848

C	-2.33946	2.30818	1.63382
C	-2.87886	2.30573	0.28355
C	-1.95295	3.03115	-0.56787
C	-1.71718	2.59855	-1.8727
C	-0.36618	2.59277	-2.4028
C	-0.21007	1.42283	-3.23508
C	1.00326	0.72743	-3.22954
C	3.3054	-1.19881	0.22627
C	-0.36618	-2.59277	-2.4028
C	0.70345	-3.01701	-1.60872
C	0.45692	-3.47261	-0.24766
C	-0.84604	-3.47917	0.25931
C	-1.95295	-3.03115	-0.56786
C	-2.40189	-1.42271	-2.38518
C	-1.47051	-0.69725	-3.2292
C	-0.21007	-1.42283	-3.23508
C	1.00326	-0.72743	-3.22954
C	2.11129	-1.18885	-2.42171
C	1.97226	-2.33863	-1.65417
C	1.56823	-3.08321	0.58117
C	1.33859	-2.6236	1.86904
C	-0.0086	-2.60774	2.40138

C	-1.08303	-3.03652	1.61442
C	-2.33946	-2.30818	1.63383
C	-2.87886	-2.30573	0.28355
C	-3.53511	-1.17516	-0.2058
C	-3.29379	-0.72546	-1.56706
C	-1.47051	0.69725	-3.2292
C	-2.40189	1.42271	-2.38518
C	-3.29379	0.72545	-1.56706
C	-3.53511	1.17516	-0.2058
C	-3.68135	0.00000	0.63468
C	-3.15998	0.00000	1.93083
C	-2.47472	-1.1758	2.43848
C	-1.35991	-0.72801	3.25441
C	-0.154	-1.42605	3.23522
C	2.58949	-2.44843	-0.30349
C	2.02815	-1.42346	2.37812
C	-1.71718	-2.59856	-1.8727
O	3.98689	2.42271	-0.03568
O	3.98689	-2.42271	-0.03568

The xyz coordinates for the structure of $C_{60}O_2$ (*cis*-3)

Charge = 0 Multiplicity = 1

C	-0.56087	-3.39237	0.76438
C	-1.01835	-2.69034	1.97853
C	-2.14318	-1.8794	1.95659
C	-2.99765	-1.75443	0.74373
C	-2.51925	-2.49575	-0.52711
C	-1.24793	-3.26715	-0.43442
C	0.88779	-3.37931	0.76926
C	1.3356	-2.6911	1.96912
C	0.16187	-2.26855	2.7105
C	0.17424	-1.04685	3.38927
C	-2.11573	-0.59221	2.60863
C	-3.35332	-0.31275	0.61204
C	-3.35361	0.31208	-0.61184
C	-2.84861	-0.37119	-1.80658
C	-2.43696	-1.69583	-1.77586
C	-1.23974	-2.09042	-2.47455
C	-0.51438	-3.05017	-1.65716
C	0.88275	-3.05247	-1.66721
C	1.59955	-3.22226	-0.42483
C	2.47097	-1.88058	1.93035
C	3.20329	-1.71324	0.68488
C	2.77698	-2.37118	-0.46844

C	2.78207	-1.67526	-1.74529
C	1.61062	-2.09832	-2.4888
C	0.91299	-1.18115	-3.27511
C	-0.53921	-1.18136	-3.26914
C	-0.98913	0.18979	-3.337
C	-2.11587	0.59162	-2.60839
C	-2.84872	0.3701	1.80699
C	-0.56198	3.39234	-0.76434
C	-1.24895	3.26671	0.43457
C	-0.51528	3.05008	1.65701
C	0.8818	3.05292	1.66697
C	1.59847	3.22265	0.42483
C	1.3346	2.69151	-1.96909
C	0.16093	2.26868	-2.71047
C	-1.0191	2.69005	-1.97885
C	-2.14362	1.87827	-1.95666
C	-2.99814	1.75381	-0.74422
C	-2.52004	2.49526	0.52777
C	-1.2402	2.08996	2.47437
C	-0.53958	1.18092	3.26905
C	0.91282	1.18136	3.27501
C	1.61004	2.09861	2.48888

C	2.78157	1.67599	1.74511
C	2.77632	2.37208	0.46832
C	3.20275	1.71421	-0.68495
C	2.47008	1.88126	-1.93032
C	0.17368	1.0467	-3.38923
C	1.35509	0.20065	-3.34964
C	2.48073	0.60838	-2.63151
C	3.20863	-0.34664	-1.81591
C	3.65312	0.33633	-0.61212
C	3.65331	-0.33527	0.61211
C	3.2087	0.34754	1.81587
C	2.48106	-0.60769	2.63135
C	1.3552	-0.20035	3.34962
C	-2.43692	1.69505	1.77608
C	-0.98908	-0.18987	3.33697
C	0.88655	3.37972	-0.76938
O	-3.72308	-2.82396	0.15288
O	-3.72346	2.82307	-0.15268

The xyz coordinates for the structure of $C_{60}O_2$ (*e*)

Charge = 0 Multiplicity = 1

C	0.23554	2.56308	2.29968

C	1.62877	2.08008	2.30205
C	2.44629	2.25752	1.19523
C	1.98653	3.02426	0.
C	0.53305	3.5269	0.
C	-0.30287	3.21243	1.19361
C	-0.56091	1.59884	3.01505
C	0.30668	0.52309	3.46542
C	1.65593	0.82135	3.0215
C	2.48958	-0.21997	2.59743
C	3.26481	1.16973	0.7287
C	2.44629	2.25752	-1.19523
C	1.62877	2.08008	-2.30205
C	0.23554	2.56308	-2.29968
C	-0.30287	3.21243	-1.19361
C	-1.62701	2.88564	-0.73789
C	-1.62701	2.88564	0.73789
C	-2.42044	1.99448	1.45199
C	-1.86046	1.31041	2.57943
C	-0.15539	-0.79128	3.4744
C	-1.50632	-1.09108	3.02605
C	-2.34303	-0.06396	2.58527
C	-3.21356	-0.25694	1.45309

C	-3.35897	1.05937	0.76818
C	-3.35897	1.05937	-0.76818
C	-2.42044	1.99448	-1.45199
C	-1.86046	1.31041	-2.57943
C	-0.56091	1.59884	-3.01505
C	3.26481	1.16973	-0.7287
C	0.70958	-1.87152	-3.03193
C	2.00762	-1.5911	-2.60193
C	2.53997	-2.2589	-1.4264
C	1.75297	-3.17713	-0.72778
C	0.40446	-3.46584	-1.17608
C	-1.47832	-2.342	-2.30745
C	-1.50632	-1.09108	-3.02605
C	-0.15539	-0.79128	-3.4744
C	0.30668	0.52309	-3.46542
C	1.65593	0.82135	-3.0215
C	2.48958	-0.21997	-2.59743
C	3.35042	-1.29975	-0.69703
C	3.35042	-1.29975	0.69703
C	2.53997	-2.2589	1.4264
C	1.75297	-3.17713	0.72778
C	0.40446	-3.46584	1.17608

C	-0.43264	-3.6413	0.00000
C	-1.74569	-3.1706	0.00000
C	-2.28569	-2.51058	-1.1766
C	-2.34303	-0.06396	-2.58527
C	-3.21356	-0.25694	-1.45309
C	-3.16398	-1.44492	-0.73838
C	-3.16398	-1.44492	0.73838
C	-2.28569	-2.51058	1.1766
C	-1.47832	-2.342	2.30745
C	-0.10778	-2.82786	2.30685
C	0.70958	-1.87152	3.03193
C	2.00762	-1.5911	2.60193
C	3.31303	-0.04207	-1.42438
C	3.31303	-0.04207	1.42438
C	-0.10778	-2.82786	-2.30685
O	1.65154	4.4063	0.00000
O	-4.48888	1.45759	0.00000

The xyz coordinates for the structure of $C_{60}O_2$ (*trans*-4)

Charge = 0 Multiplicity = 1

C	-0.60389	0.45963	3.49148
C	-1.82954	-0.22599	3.04025

C	-1.79483	-1.54945	2.62506
C	-0.54413	-2.36149	2.69689
C	0.73271	-1.64372	3.16726
C	0.61658	-0.19833	3.51215
C	-0.67445	1.83271	3.03062
C	-1.91987	2.00553	2.30241
C	-2.62864	0.73824	2.3092
C	-3.35324	0.34526	1.17737
C	-2.49541	-1.938	1.42802
C	-0.46259	-3.12964	1.42159
C	0.73296	-3.26908	0.72257
C	1.95365	-2.57876	1.17768
C	1.94667	-1.77903	2.31045
C	2.60758	-0.49627	2.28672
C	1.79712	0.46569	3.01588
C	1.74505	1.79656	2.59202
C	0.47924	2.49425	2.59804
C	-1.96368	2.82744	1.17535
C	-0.76068	3.51143	0.7251
C	0.43606	3.35062	1.42487
C	1.68318	3.18254	0.69842
C	2.4949	2.2197	1.42236

C	3.27527	1.29716	0.72517
C	3.33578	-0.08673	1.16777
C	3.37383	-0.937	0.
C	2.68686	-2.15874	0.
C	-1.67668	-2.89646	0.69975
C	0.73271	-1.64372	-3.16726
C	-0.54413	-2.36149	-2.69689
C	-1.79483	-1.54945	-2.62506
C	-1.82954	-0.22599	-3.04025
C	-0.60389	0.45963	-3.49148
C	1.79712	0.46569	-3.01588
C	2.60758	-0.49627	-2.28672
C	1.94667	-1.77903	-2.31045
C	1.95365	-2.57876	-1.17768
C	0.73296	-3.26908	-0.72257
C	-0.46259	-3.12964	-1.42159
C	-2.49541	-1.938	-1.42802
C	-3.28469	-1.02446	-0.7271
C	-3.35324	0.34526	-1.17737
C	-2.62864	0.73824	-2.3092
C	-1.91987	2.00553	-2.30241
C	-0.67445	1.83271	-3.03062

C	0.47924	2.49425	-2.59804
C	1.74505	1.79656	-2.59202
C	3.33578	-0.08673	-1.16777
C	3.27527	1.29716	-0.72517
C	2.4949	2.2197	-1.42236
C	1.68318	3.18254	-0.69842
C	0.43606	3.35062	-1.42487
C	-0.76068	3.51143	-0.7251
C	-1.96368	2.82744	-1.17535
C	-2.70981	2.41313	0.
C	-3.39439	1.19814	0.
C	-1.67668	-2.89646	-0.69975
C	-3.28469	-1.02446	0.7271
C	0.61658	-0.19833	-3.51215
O	0.13299	-2.70532	3.89898
O	0.13299	-2.70532	-3.89898

The xyz coordinates for the structure of $C_{60}O_2$ (*trans*-3)

Charge = 0 Multiplicity = 1

C	-2.99771	0.10821	-1.90986
C	-2.30446	-1.084	-2.42832
C	-2.16659	-2.22594	-1.65386

C	-2.78488	-2.32997	-0.29923
C	-3.50639	-1.08216	0.24429
C	-3.5292	0.12636	-0.6282
C	-2.30419	1.27662	-2.41816
C	-1.19668	0.82774	-3.24082
C	-1.19334	-0.62641	-3.24047
C	0.02257	-1.31646	-3.23153
C	-0.89616	-2.90571	-1.60615
C	-1.76396	-2.96230	0.58417
C	-1.53133	-2.507	1.87236
C	-2.22213	-1.31427	2.39429
C	-3.12552	-0.61292	1.60799
C	-3.07779	0.826	1.57651
C	-3.325	1.27719	0.21477
C	-2.67751	2.41575	-0.27038
C	-2.15651	2.41576	-1.61781
C	0.00726	1.53075	-3.24237
C	0.15907	2.7135	-2.40836
C	-0.90163	3.14667	-1.61158
C	-0.65095	3.59988	-0.25262
C	-1.75084	3.14666	0.57859
C	-1.50997	2.71321	1.88213

C	-2.18973	1.53208	2.3929
C	-1.2695	0.80569	3.23335
C	-1.28	-0.59305	3.22773
C	-0.65345	-3.34834	-0.24976
C	2.30473	-1.08576	2.42759
C	2.16685	-2.22694	1.65198
C	2.78566	-2.32939	0.29774
C	3.50654	-1.08127	-0.24599
C	3.52934	0.12627	0.62821
C	2.30385	1.27476	2.41923
C	1.19647	0.82496	3.24156
C	1.19345	-0.62918	3.24006
C	-0.0223	-1.31957	3.23042
C	-0.17887	-2.48847	2.39587
C	0.8968	-2.90717	1.60344
C	1.76523	-2.96133	-0.58685
C	1.53197	-2.50466	-1.87444
C	2.22241	-1.31145	-2.39577
C	3.12544	-0.61038	-1.60879
C	3.07754	0.82842	-1.57572
C	3.32473	1.27808	-0.21346
C	2.67692	2.41605	0.27263

C	2.15585	2.41463	1.62003
C	-0.00766	1.52763	3.24377
C	-0.1598	2.71109	2.41083
C	0.90083	3.14529	1.61452
C	0.6501	3.59974	0.25598
C	1.75009	3.14757	-0.57565
C	1.50923	2.71543	-1.87963
C	2.18929	1.53496	-2.39152
C	1.26929	0.80911	-3.23266
C	1.28007	-0.58963	-3.2284
C	0.65457	-3.34845	0.24655
C	0.1795	-2.48605	-2.398
C	2.99757	0.10697	1.90974
O	-4.18202	-2.30104	-0.03732
O	4.18263	-2.30004	0.03516

The xyz coordinates for the structure of $C_{60}O_2$ (*trans*-2)

Charge = 0 Multiplicity = 1

C	3.20434	1.48765	0.60677
C	2.95582	0.74832	1.85889
C	2.96514	-0.63975	1.88406
C	3.31699	-1.4413	0.67577

C	3.5807	-0.66731	-0.63091
C	3.45755	0.81795	-0.58114
C	2.34219	2.65317	0.60761
C	1.57053	2.64923	1.83765
C	1.94936	1.47481	2.60564
C	0.97805	0.78459	3.34035
C	1.93049	-1.34864	2.59218
C	2.34394	-2.56844	0.61699
C	1.76567	-2.96985	-0.57716
C	2.01921	-2.22972	-1.82919
C	2.84339	-1.11342	-1.84759
C	2.43436	0.06724	-2.56781
C	2.8066	1.24241	-1.79554
C	1.98621	2.37413	-1.81099
C	1.74893	3.09589	-0.58003
C	0.24669	3.08968	1.83544
C	-0.36893	3.54357	0.59793
C	0.36927	3.5457	-0.58496
C	-0.24638	3.09652	-1.82403
C	0.75935	2.37516	-2.58562
C	0.40075	1.2509	-3.32781
C	1.25752	0.07578	-3.32231

C	0.41521	-1.09667	-3.32949
C	0.78582	-2.22261	-2.58904
C	1.54736	-2.5208	1.818
C	-2.3445	-2.56581	-0.62613
C	-1.7662	-2.97148	0.5662
C	-2.01966	-2.23646	1.82124
C	-2.84353	-1.12004	1.84369
C	-3.58123	-0.66968	0.62885
C	-2.96474	-0.63236	-1.88647
C	-1.93066	-1.33879	-2.59707
C	-1.54751	-2.51373	-1.82729
C	-0.21891	-2.94381	-1.82632
C	0.38088	-3.40051	-0.59437
C	-0.3812	-3.40267	0.58192
C	-0.78619	-2.23218	2.58088
C	-0.41535	-1.10898	3.32534
C	-1.25737	0.06347	3.32234
C	-2.43428	0.05791	2.56791
C	-2.80646	1.23588	1.79989
C	-3.45735	0.81595	0.5843
C	-3.20395	1.49009	-0.60119
C	-2.95562	0.75572	-1.85631

C	-0.96743	-0.64851	-3.33642
C	-0.97789	0.79696	-3.33731
C	-1.94921	1.48463	-2.60011
C	-1.57023	2.6563	-1.82777
C	-2.34217	2.65585	-0.59792
C	-1.7487	3.09398	0.59144
C	-1.9859	2.36747	1.81949
C	-0.75903	2.36554	2.59425
C	-0.4005	1.2386	3.33235
C	0.21845	-2.95059	1.81531
C	0.96741	-0.66109	3.33396
C	-3.31689	-1.43803	-0.68118
O	4.58476	-1.42443	0.03271
O	-4.58494	-1.42392	-0.03808

The xyz coordinates for the structure of $C_{60}O_2$ (*trans*-1)

Charge = 0 Multiplicity = 1

C	2.60921	0.7399	2.29778
C	2.60921	-0.7399	2.29778
C	3.0535	-1.45244	1.19572
C	3.62739	-0.76961	0.00000
C	3.62739	0.76961	0.00000

C	3.0535	1.45244	1.19572
C	1.42881	1.17894	3.01273
C	0.69717	0.00000	3.45308
C	1.42881	-1.17894	3.01273
C	0.72666	-2.30857	2.59567
C	2.29913	-2.58814	0.73009
C	3.0535	-1.45244	-1.19572
C	2.60921	-0.7399	-2.29778
C	2.60921	0.7399	-2.29778
C	3.0535	1.45244	-1.19572
C	2.29913	2.58814	-0.73009
C	2.29913	2.58814	0.73009
C	1.17188	3.03578	1.42266
C	0.72666	2.30857	2.59567
C	-0.69717	0.00000	3.45308
C	-1.42881	1.17894	3.01273
C	-0.72666	2.30857	2.59567
C	-1.17188	3.03578	1.42266
C	0.00000	3.48744	0.6942
C	0.00000	3.48744	-0.6942
C	1.17188	3.03578	-1.42266
C	0.72666	2.30857	-2.59567

C	1.42881	1.17894	-3.01273
C	2.29913	-2.58814	-0.73009
C	-1.42881	-1.17894	-3.01273
C	-0.72666	-2.30857	-2.59567
C	-1.17188	-3.03578	-1.42266
C	-2.29913	-2.58814	-0.73009
C	-3.0535	-1.45244	-1.19572
C	-2.60921	0.7399	-2.29778
C	-1.42881	1.17894	-3.01273
C	-0.69717	0.00000	-3.45308
C	0.69717	0.00000	-3.45308
C	1.42881	-1.17894	-3.01273
C	0.72666	-2.30857	-2.59567
C	0.00000	-3.48744	-0.6942
C	0.0000	-3.48744	0.6942
C	-1.17188	-3.03578	1.42266
C	-2.29913	-2.58814	0.73009
C	-3.0535	-1.45244	1.19572
C	-3.62739	-0.76961	0.00000
C	-3.62739	0.76961	0.00000
C	-3.0535	1.45244	-1.19572
C	-0.72666	2.30857	-2.59567

C	-1.17188	3.03578	-1.42266
C	-2.29913	2.58814	-0.73009
C	-2.29913	2.58814	0.73009
C	-3.0535	1.45244	1.19572
C	-2.60921	0.7399	2.29778
C	-2.60921	-0.7399	2.29778
C	-1.42881	-1.17894	3.01273
C	-0.72666	-2.30857	2.59567
C	1.17188	-3.03578	-1.42266
C	1.17188	-3.03578	1.42266
C	-2.60921	-0.7399	-2.29778
O	4.82305	0.00000	0.00000
O	-4.82305	0.00000	0.00000

Reference

1 Only two peaks for the 2 sp^3-carbons and four peaks for the half-intensity sp^3-carbons of the C_{60} skeleton were given in the literature: Balch AL, Costa DA, Noll BC, Olmstead MM. 1995 Oxidation of buckminsterfullerene with *m*-chloroperoxybenzoic acid, Characterization of a C_s isomer of the diepoxide $C_{60}O_2$. *J. Am. Chem. Soc.* **117**, 8926–8931. (doi:10.1021/ja00140a005)
